# ONT-Based Alternative Assemblies Impact on the Annotations of Unique versus Repetitive Features in the Genome of a Romanian Strain of *Drosophila melanogaster*

**DOI:** 10.3390/ijms232314892

**Published:** 2022-11-28

**Authors:** Alexandru Marian Bologa, Ileana Stoica, Attila Cristian Ratiu, Nicoleta Denisa Constantin, Alexandru Al. Ecovoiu

**Affiliations:** Department of Genetics, Faculty of Biology, University of Bucharest, 060101 Bucharest, Romania

**Keywords:** *Drosophila melanogaster*, nanopore sequencing, MinION, ONT, de novo genome assembly, natural transposons

## Abstract

To date, different strategies of whole-genome sequencing (WGS) have been developed in order to understand the genome structure and functions. However, the analysis of genomic sequences obtained from natural populations is challenging and the biological interpretation of sequencing data remains the main issue. The MinION device developed by Oxford Nanopore Technologies (ONT) is able to generate long reads with minimal costs and time requirements. These valuable assets qualify it as a suitable method for performing WGS, especially in small laboratories. The long reads resulted using this sequencing approach can cover large structural variants and repetitive sequences commonly present in the genomes of eukaryotes. Using MinION, we performed two WGS assessments of a Romanian local strain of *Drosophila melanogaster*, referred to as Horezu_LaPeri (Horezu). In total, 1,317,857 reads with a size of 8.9 gigabytes (Gb) were generated. Canu and Flye de novo assembly tools were employed to obtain four distinct assemblies with both unfiltered and filtered reads, achieving maximum reference genome coverages of 94.8% (Canu) and 91.4% (Flye). In order to test the quality of these assemblies, we performed a two-step evaluation. Firstly, we considered the BUSCO scores and inquired for a supplemental set of genes using BLAST. Subsequently, we appraised the total content of natural transposons (NTs) relative to the reference genome (ISO1 strain) and mapped the mdg1 retroelement as a resolution assayer. Our results reveal that filtered data provide only slightly enhanced results when considering genes identification, but the use of unfiltered data had a consistent positive impact on the global evaluation of the NTs content. Our comparative studies also revealed differences between Flye and Canu assemblies regarding the annotation of unique versus repetitive genomic features. In our hands, Flye proved to be moderately better for gene identification, while Canu clearly outperformed Flye for NTs analysis. Data concerning the NTs content were compared to those obtained with ONT for the *D. melanogaster* ISO1 strain, revealing that our strategy conducted to better results. Additionally, the parameters of our ONT reads and assemblies are similar to those reported for ONT experiments performed on various model organisms, revealing that our assembly data are appropriate for a proficient annotation of the Horezu genome.

## 1. Introduction

The genome contains the genetic information necessary for a given species to function optimally in its environment. Coding and non-coding regions, mutations, structural variants, as well as other genomic entities such as transposable elements and regulatory elements can be identified through various molecular and bioinformatics analyses. Sequencing experiments generate large genomic data, thus making the bioinformatics analysis a real challenge. Currently, the main difficulties are caused by the limitations of current methods of data analysis, as well as the complexity of handling high-throughput data.

Assembling genomic sequences is one of the most important steps in genomics [1]. Long reads generated by Oxford Nanopore Technologies (ONT) sequencing are useful for analyzing repetitive regions and structural variants in genomes and contribute to the quality and the completeness of an assembly. However, sequences generated by this type of technology are reported to have a relatively high error rate, which can be at least partially corrected before the assembly process [2,3].

There are currently two distinct strategies used for de novo assembly of long sequences: a correction step performed directly on the assembly or a read correction step followed by their assembly [2]. Some de novo sequence assemblers, such as Flye [4] and Shasta [5] start by generating the assembly of uncorrected reads and then refine the genome assembly. Conversely, tools such as Canu [6] initiate sequences’ correction and then assemble the corrected reads. Both assembling strategies have strengths and drawbacks in terms of computational requirements, working time, contiguity and accuracy of the resulting assembly.

In this study, we describe and compare alternative genome assemblies of long reads generated by nanopore genome sequencing of a Romanian local strain of *Drosophila melanogaster* (the fruit fly), named Horezu_LaPeri (Horezu). We used multiple bioinformatics applications dedicated to the assembly of long reads such as Flye [4], Canu [6], minimap2 [7] and NGMLR [8] and then compared the results obtained with either unfiltered or quality filtered read datasets. The strategy of using unfiltered data proved to be more proficient for the annotation of NTs.

## 2. Results

### 2.1. Nanopore Sequencing

We performed two nanopore sequencing runs, i.e., Run_1 with 173 FAST5 files, and Run_2 with 158 FAST5 files, respectively. As a result of the basecalling process, a total of 688,560 reads (5 Gb) were generated in Run_1 and 629,570 reads (4.1 Gb) in Run_2. Overall, the mean read length of Run_1 was 3548 nucleotides (nt) and 3171 nt for Run_2. The longest read from Run_1 has 121,786 nt and a Phred quality score (q) of 3.7, while the longest read from Run_2 reads has 112,857 nt with q = 6.8. The highest mean q is 19.1 for a 1853 nt read in Run_1, and 18.1 for a 2006 nt read in Run_2. Regarding the overall quality scores, only 86.43% reads from Run_1 and 65.96% reads from Run_2 passed the quality filter of EPI2ME platform and have a q > 7.

The mean q of Run_1 reads was significantly higher than that of Run_2 (10.8 compared to 8.1). Various sequencing statistics of the raw FASTQ files generated by both sequencing runs were calculated using NanoPlot [9] and are summarized in Table 1.

To generate assemblies, we used coalesced Run_1 and Run_2 data. The two datasets were concatenated in a single FASTQ file submitted to Porechop version 0.2.4 [10] for Rapid adapter removal. The resulting collection of reads was filtered with NanoFilt [9], and the sequences with q < 10 were discarded.

Therefore, two new datasets were generated:

1. Data set I, represented by a concatenated FASTQ file that contains all the trimmed reads;
2. Data set II, where the concatenated FASTQ file contains only trimmed and filtered reads.

The statistical parameters describing Data set I and Data set II were obtained with NanoPlot and are detailed in Table 2.

### 2.2. De Novo Assembly

Starting from Data set I and Data set II, we generated four de novo assemblies using Canu [6] and Flye [4]. The resulting assemblies are symbolized by Canu—Data set I, Canu—Data set II, Flye—Data set I, and Flye—Data set II and were assessed for quality with QUAST-LG [11]. If a reference genome is available, QUAST-LG computes the assembly completeness (fraction of the reference genome), correctness (% errors in the assembly) and contiguity (number of fragments and their length), as well as the generic N50 and NG50 metrics. Assemblies based on Data set II have better statistics only for the largest contig, N50, NG50 and number of possible natural transposons (NTs) (Table 3).

**Figure 1:**
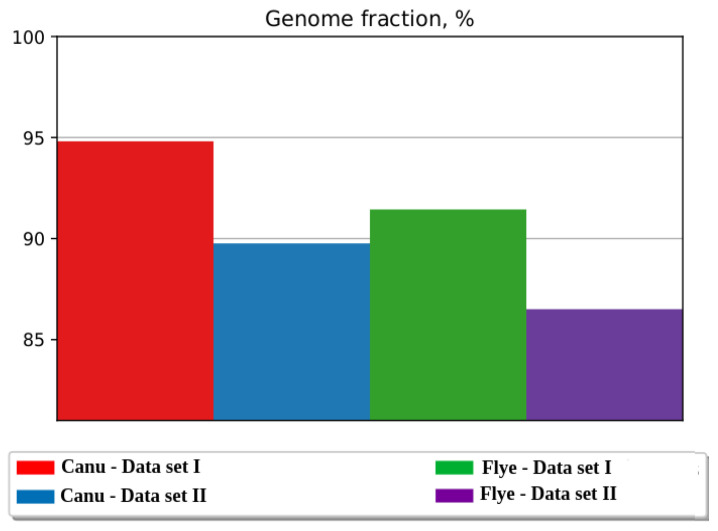
Coverage percentage of the *D. melanogaster* reference genome (r6.39) for the contigs obtained with Canu and Flye from Data set I and Data set II (source: QUAST-LG).

The coverage percentage of *D. melanogaster* reference genome (r6.39) has the highest value for Canu—Data set I, while the lower value was obtained for Flye—Data set II assembly (Figure 1).

The highest values for genome fraction coverage were obtained for Canu assemblies, but the overall quality of these assemblies is lower relative to the assemblies obtained with Flye (Table 3). For example, statistics such as N50, the largest contig, the number of misassembled or unaligned contigs are better for Flye assemblies.

**Figure 2:**
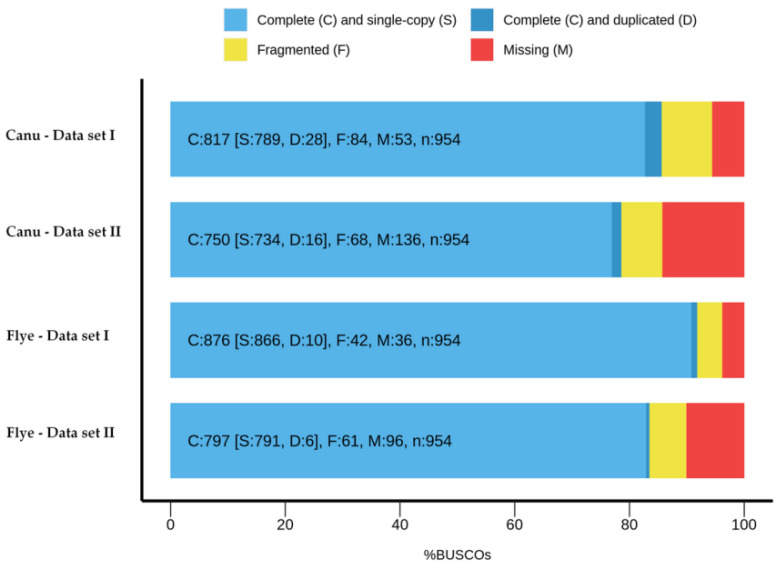
BUSCO assessment of the four de novo genome assemblies. Bar charts show gene proportions classified as: complete (C) as single-copy (S, light blue) or duplicated (D, dark blue); fragmented (F, yellow) and missing (M, red). Inside the light blue bars the number of genes falling into each category (BUSCO scores) is displayed.

Each assembly was scanned for 954 highly conserved universal single-copy orthologues (USCOs) using BUSCO equipped with metazoa_odb10 [12]. The best result was obtained for Flye—Data set I assembly (Figure 2).

BUSCO scores presented in Figure 2 indicate that the metazoan gene set was well represented in our four assemblies. The Flye—Data set I assembly contains the minimum number of fragmented or missing USCOs (42 and 36, respectively) and has the best scores for complete USCOs, either in single-copy (866) or duplicated (10).

For a supplementary assessment of the quality of these assemblies, we performed a BLAST [13] screening in order to check for the presence of a predefined set of 53 genes (Table S1) involved in *D. melanogaster* immunity. These genes pertain to Toll, Imd and Imd-JNK pathways, except the *γCOP* gene, which impacts the innate immune response of *D. melanogaster* [14]. Out of them, *sphe* was present only in Flye—Data set I assembly, while *krz* was missing in both Flye assemblies. Alternatively, the *lic* gene was absent only in the assemblies generated with filtered data. The quality of the alignments, indicated by percent identity and mismatch number, was very similar for almost any given gene in all of the assemblies (Table S2). Considerable mismatches were found for *ben* gene in Flye—Data set II assembly and for *hep* in both assemblies generated with Data set II. For *Oamb*, *Plc21C* and *Gprk2* genes we obtained fragmented BLAST alignments, therefore they were not considered for further inquiries. We found >95% identities for 44 genes in each of the assemblies. Remarkably, *Drs*, *Drsl1*, *Mtk* and *wntD* had 100 percent identity in Flye—Data set II assembly.

Considering the results obtained with BLAST on Canu—Data set I assembly, we plotted the genes according to their length, alignment length and number of mismatches (Figure S1). As expected, the number of mismatches increases in the gene versus assembly alignments with the gene length.

The characterization of a natural population in terms of the total content of NTs is an important aspect in genomics and evolutionary studies [15,16,17]. The percentage and mapping of particular NTs insertions are key aspects in the qualitative and quantitative analysis of a genome. We analyzed our de novo assemblies of Horezu strain versus a minimap/miniasm de novo assembly of ISO1 strain [18], symbolized minimap/miniasm—ISO1, since all of these assemblies are constructed exclusively from ONT reads. Although reads from two different sequencing technologies (long and short reads) may be combined in order to improve the assembly quality [18], we inquired if using only ONT data is a reliable approach for Horezu genome analysis.

The comparative analysis considered the evaluation of the total content in NTs with RepeatMasker version 4.1.2 (relying on rmblastn version 2.10.0+ and CONS-Dfam_withRBRM_3.3) [19]. We also mapped mdg1 retroelement (an LTR transposon from the Gypsy group) in these assemblies using Genome ARTIST (GA_v2) software [20].

The rationale of our comparative analysis was to identify the best alternative assembly to be used for mapping and annotation of repetitive sequences as NTs in *D. melanogaster* genome. As presumed, the global approach performed with RepeatMasker (Table 4) revealed that the Canu—Data set I assembly is by far the most relevant one (Bases masked = 26.83%; Retroelements = 18.93%; DNA transposons = 1.39%). Data were as expected since this assembly was obtained with unfiltered reads; therefore, most of the repetitive sub-sequences (many of them prone to be NTs) are kept in the assembly. The highest percentage of NTs in Horezu strain was revealed by the analysis of the Canu—Data set I assembly (18.93 + 1.39 = 20.32%), compared to the percentages obtained for Canu—Data set II (18.46 + 1.39 = 19.85%), Flye—Data set I (8.65 + 1.01 = 9.66%), Flye—Data set II (8.17 + 1.01 = 9.18%) and the minimap/miniasm—ISO1 (12.49 + 1.12 = 13.61%). The differences between the two Canu assemblies seem to be minor relative to the total content in NTs, but they are significant for some NTs families instead. We noticed that Jockey (1727 versus 1434), Copia (772 versus 671) and Gypsy (10,436 versus 8912) families have a higher number of elements in the Canu—Data set I assembly. Total NTs content values computed for Canu assemblies are in accordance with the total NTs content of *D. melanogaster* reference genome which was estimated at ~20% [21].

A complementary qualitative test was performed by individually mapping a particular retrotransposon in Horezu strain versus ISO1 strain, in order to detect minute similitudes and differences between two NTs genomic landscapes. We presumed that a genome assembly obtained from unfiltered reads would be more complete, offering better results of retrotransposons mapping comparative to the filtered alternatives. In order to test this assumption, we considered mdg1 retroelement, since it is potentially active and may occur as full-length copies in the genome of *D. melanogaster* [22,23]. The mapping was performed with GA_v2 tool, using a strategy described elsewhere [20]. The majority of mdg1 insertions were mapped at nucleotide level relative to the *D. melanogaster* reference genome (r6.48), either in intergenic regions or in specific genes (Tables S3–S7). Some insertions were found in all Horezu assemblies, such as *Pzl* insertion, while others are assembly specific. The insertion in *pum* is detectable only in Canu assemblies, the insertion in *heph* is found exclusively in the assemblies generated with the Data set II and *Rbp1* insertion is specific for Canu—Data set I assembly. These data reveal that no de novo assembly procedure offers complete or unambiguous results. Regarding the number of mapped mdg1 insertions, the Canu assemblies appear to harbor most of them. Canu—Data set I assembly contains the highest number of mapped mdg1 insertions (44), in accordance with our starting hypothesis, that using unfiltered reads is appropriate for NTs mapping projects. On the other hand, we mapped 11 mdg1 insertions for each of the Flye assemblies. Conversely, the minimap/miniasm—ISO1 contains 17 mdg1 insertions, relative to the 43 mdg1 insertions annotated for the *D. melanogaster* reference genome (r6.48).

The comparative results for Canu—Data set I, Canu—Data set II, Flye—Data set I, Flye—Data Set II and minimap/miniasm—ISO1 assemblies are summarized in Table 5.

### 2.3. Guided Assembly versus the Reference Genome of D. melanogaster

**Figure 3:**
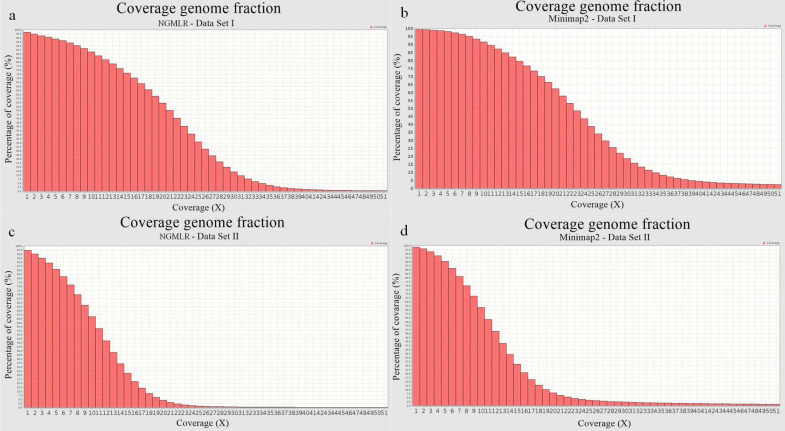
Genome Fraction Coverage (*y* axis) and coverage level (*x* axis) obtained with NGMLR (**a**,**c**) and minimap2 (**b**,**d**) by mapping the two datasets (Data set I—a and b; and Data set II—c and d) to the *D. melanogaster* reference genome (r6.39) (source: Qualimap).

The guided assembly versus the *D. melanogaster* reference genome (r6.39) was performed using minimap2 [7] and NGMLR [8] applications with both datasets. The resulting files were evaluated with the Qualimap [24] and BAMstats [25] quality assessment programs. Four BAM files were compared in terms of assembly quality. Following the Qualimap and BAMstats analyses, we found that the minimap2—Data set I assembly had the highest coverage percentage (Figure 3).

As shown in Figure 3, the coverage percentage decreases with the depth of coverage (X). When considering a mean coverage of 10, there is a dramatic decrease of 30% for the genome fraction coverage if Data set II is used instead of Data set I.

As presumed, the value of genome coverage for assemblies of Data set II is lower compared to Data set I assemblies, since Data set II contains fewer reads but with higher quality scores. Accordingly, the general error rate of a selected assembly (indicated by the ratio between matches and mismatches) is higher when using Data set I. Table 6 lists the statistical parameters obtained with Qualimap for the four assemblies.

Overall, the minimap2—Data set I assembly appears to be reliable, at least in terms of mean coverage and genome fraction coverage. The coverage values and the number of aligned reads for each chromosome were computed for the four assemblies using the BAMstats application (Table S8).

## 3. Discussion

Our study is the first sequencing project of a Romanian local natural strain of *D. melanogaster*, named Horezu and collected from Horezu region. We evaluated if a rapid ONT sequencing kit designed for fast library preparation without a ligation step is appropriate to generate collections of long reads suitable for a good quality genome assembly. We were also concerned if genomic assemblies generated by various methods are suitable for accurate annotation of various genes and NTs. Our results highlight that the qualitatively unfiltered sequencing reads are of adequate quality when searching either for sequences of predefined sets of genes or for NTs mapping. In addition, de novo and guided assembly steps performed using the unfiltered reads revealed several advantages in terms of coverage and assembly completeness.

Using Data set I for de novo assembly, we obtained a genome fraction coverage of 94.8% with Canu and 91.44% with Flye, respectively (Table 3). Interestingly, the Flye assembly does not output the highest genome coverage, but it generates better values for key qualitative parameters, such as the largest contig, the longest alignment or the N50 score. These characteristics could bring an advantage for the identification and analysis of genes in Horezu genome. Conversely, searching for NTs revealed that the Canu assembly considerably outperforms the Flye one according to the results of RepeatMasker and mdg1 assessments.

The Canu—Data set II and Flye—Data set II assemblies provided *D. melanogaster* reference genome coverages of 89.7% and 86.5%. Important parameters, such as the overall error rate during assembly, the lower number of misaligned bases and the reduced number of partially misaligned contigs, displayed better values for the assemblies compiled with Data set II. These distinctive features are adequate for identification of structural variants or genes. Confirmatory, Flye—Data set II assembly harbors the maximum number of genes having 100 percent identity with the corresponding reference sequences. Conversely, the Canu—Data set II assembly is a better option than the Fly—Data set II assembly for NTs mapping.

As an overall quality control, we mapped both genes and NTs in the four distinct assemblies. The best BUSCO score was achieved for Flye—Data set I assembly that has 876 USCOs (91.8%) detected in at least one copy (Figure 2). The assemblies obtained with Data set I provided better BUSCO scores than those generated using Data set II. For example, Canu—Data set II assembly allows for detection of only 750 complete USCOs. Since BUSCO assessment can provide false-positive results [26], we tested the assembling quality using BLAST and a supplemental set of genes involved in innate immunity (Toll and Imd-JNK pathways genes). The majority of the genes displayed similarity scores of over 95% in each assembly, but for a few genes minor quality issues were detected in the assemblies obtained with filtered reads. Globally, there are only small differences among the four assemblies of Horezu strain, as shown in Table S2. We conclude that searching for genes in assemblies compiled from filtered reads provides some quality improvements.

Regarding the NTs mapping, the differences between Canu and Flye assemblies are obvious. The results obtained with RepeatMasker indicate that Canu—Data set I assembly offers the best values for every considered NTs category, while the Flye assemblies are outperformed by the Canu ones. Canu—Data set I assembly has a total NTs content representing approximatively 20.32% of the Horezu genotype. Since the estimated total NTs content of the *D. melanogaster* reference genome is ~20% [21], it appears that the stand alone ONT sequencing is very reliable for the analysis of transposons in the Drosophilidae genomes. The differences between Canu assemblies are not very evident, but Canu—Data set I allows for a better mapping for a selection of NTs, such as Gypsy and Copia transposon families according to RepeatMasker (10,436 versus 8912 copies and 772 versus 671 copies, respectively). As a complementary approach assessing minute quality differences, we mapped mdg1 retroelement and, as expected, the results revealed that Canu—Data set I assembly is the best option for this purpose.

The NTs analyses were also performed on the minimap/miniasm—ISO1 assembly [18], using RepeatMasker and GA_v2. The comparative analysis of minimap/miniasm—ISO1 and Horezu assemblies regarding NTs detection reveals that Canu—Data set I assembly is the most appropriate one for this purpose. The total content of NTs identified in Canu—Data set I (20.32%) is substantially higher as compared to minimap/miniasm—ISO1 (13.61%). Accordingly, the number of mdg1 copies identified with GA_v2 in the respective assemblies is 44 versus 17.

Therefore, care should be taken when considering what sequencing data are to be used with de novo assemblers, such as Canu and Flye. Adjustments of the assembly strategy paradigm might be considered in accordance with the research objectives.

The analysis of Horezu-guided assemblies indicated that minimap2 was the most efficient one for mapping reads to the reference genome for both datasets. The percentage of aligned reads, the average coverage value and the average alignment quality had the best values when using minimap2 (Table 5). Additionally, the BAMstats analysis revealed that the averages of the coverage values for each chromosome are higher for the assemblies generated with minimap2 (Table S8).

Next generation sequencing technologies are used on a large scale in whole-genome sequencing (WGS) projects, but short reads fail to cover the entire genome, often leaving gaps or producing assembly errors in repetitive regions [27,28]. Instead, long-read sequencing technologies have been tested for sequencing of large genomes, mainly those of model organisms, in order to simplify genome assembly and to resolve low-complexity regions [29,30]. For example, using ONT for genome sequencing of the experimental model *Arabidopsis thaliana*, Debladis et al. [31] generated a number of 118,554 reads with a minimum length of 6 nt, a maximum of 691,915 nt and a median of 4.6 kb. Even with a low level of coverage, their sequencing data allowed the identification of transposon insertions such as LTR retroelements and DNA transposons CACTA and CAC1. In a similar study, Michael et al. [32] sequenced genomic DNA from *Arabidopsis thaliana* and obtained 300,053 sequencing reads with an average read length of 11.4 kb. WGS experiments using nanopore sequencing were also performed on pea (*Pisum sativum*) and approximately 33.2 million reads with an N50 read length of 15.5 kb, totaling 262.1 Gb of data were obtained. After de novo assembly, a number of 117,981 contigs (3.3 Gb) were generated, with an N50 value of 51.2 kb and a BUSCO score of 51% [33]. For zebrafish (*Danio rerio*) the long-read sequencing of its genome produced sequences with N50 = 15 Kb and a value of 464,751 nt for the longest read. Assembly generated with Canu (1.42 Gb) showed a coverage of the reference genome of 90.8%, while the assembly produced with miniasm (1.39 Gb) had 88% coverage [34]. The *Caenorhabditis elegans* genome was recently recompleted in a sequencing experiment using ONT that generated a number of 225,835 raw reads. After filtering according to quality score, 166,198 reads were obtained with an average length of 16,413, minimum length of 15 nt and maximum read length of 336,266 nt [35]. In a different study performed on *C. elegans* VC2010 wild-type strain [36], combined data from three flow cells, consisting of 1,116,324 reads, revealed an average read length ranging from 13 kb to 20 kb, with a maximum of 134 kb. Raw reads were filtered according to quality score (q >10) and size (>1 kb), improving sequence quality but reducing the number of reads (583,466). When utilizing only q10 long reads for genome assembly, Canu generated 73 contigs, the largest contig having more than 9.9 Mb. Moreover, half of the reference genome was contained in the 10 largest contigs. In addition, the contigs were corrected with Illumina short reads, increasing sequence identity with the reference genome to 99.8% [36].

ONT was used in 2018 to sequence the genomes of 15 species of Drosophilidae [37]. A total of 23 million reads were generated, with an average read length of 4302 nt. A proportion of 76% of reads passed Albacore filter (≥7) and had an average read length of 5894 nt. Genome assembly was performed with Canu and miniasm, which had similar assembly statistics: an average contig N50 value of 4.4 Mb and average BUSCO score of 97.7% [37]. Additionally, in a study aiming to test ONT technology on *D. melanogaster* reference genome, Solares et al., generated a total of 663,784 reads with an average read length of 7122 nt. A number of 593,354 (89%) of all reads were marked as “pass” (having a quality score ≥7). A comparison between Canu and minimap/miniasm assemblies revealed a higher accuracy and completeness of the Canu assembly (contig N50 = 3.0 Mb and BUSCO score of 67.7%) [18]. In another recent study using nanopore technology for sequencing, 101 Drosophilidae species, Flye assemblies with N50 average of 10.5 Mb and a BUSCO score greater than 97% were obtained for 97/101 of them [38]. N50 values of 6.6 Mb and 5.4 Mb were obtained for contigs assembled with Canu and scaffolded with Hi-C data in a study using 713,692 and 481,640 reads for the DGRP379 and DGRP732 strains of *D. melanogaster* [39].

On average, the parameters of our ONT reads and assemblies are in the range of the values reported for the above mentioned ONT experiments and the results of the quality assessments by detection of genes and NTs are supportive. Therefore, we consider that our ONT only genome assemblies are reliable for the annotation of both unique and repetitive genomic features of Horezu strain. This approach could contribute to a more detailed analysis and understanding of the structure and evolution of *D. melanogaster* genome, as no Romanian fruit fly strain was sequenced so far.

## 4. Materials and Methods

### 4.1. Fly Stock

The fruit flies were collected in August 2018 from the location Romanii de Sus, Horezu, Vâlcea County, Romania. For isogenization, Horezu stock was maintained for about 2 years at 18 °C in standard medium-sized bottles containing culture medium based on an agar and banana recipe. Prior to sequencing, the fly stock was maintained for one day at 25 °C.

### 4.2. DNA Isolation and Quantification

To obtain long DNA fragments, we performed an adapted DNA extraction protocol previously described by Miller et al. [37].

We collected about 50 *D. melanogaster* males from the Horezu strain, which were kept at −20 °C for about an hour before DNA extraction. We used pestles to grind the chitinous layer of the cuticle of the frozen males placed in an 1.5 mL Eppendorf tube in which we added 1 mL homogenization buffer (0.1 M NaCl, 30 mM Tris-HCL, 10 mM EDTA, 0.5% Triton X).

The homogenized suspension was transferred to a 1.5 mL Eppendorf tube using a wide-bore pipette tip and the tissue debris were separated by centrifugation at 500× *g* at 4 °C for 1 min. Supernatant was then transferred into a new tube, and nuclei were pelleted by centrifugation 5 min at 2000× *g* at 4 °C. Pelleted nuclei were resuspended in 200 µL homogenization buffer. For nuclear membrane lysis, we added 1.268 mL extraction buffer (0.1 M TrisHCl, 0.1 M NaCl, 20 mM EDTA), 1.5 µL proteinase K (20 mg/mL) and 30 µL of 10% SDS. Subsequently, the tube was maintained at 37 °C for about 3 h. The nucleic acid solution was mixed with equal volumes of phenol: chloroform: isoamyl alcohol pH 8.0. We performed a succession of two homogenizations and two centrifugations at 5000× *g* for 5 min at room temperature with the transfer of the upper aqueous phase after each step. Finally, we transferred the aqueous phase to a new tube over which we added 3M sodium acetate (NaOAc) (10% *v*/*v*) and ethanol (EtOH) 97% (twice the volume of the aqueous phase).

After an overnight incubation at 4 °C, we stimulated DNA precipitation by adding 2 µL glycogen and centrifuged the solution at 14,000 rpm at 4 °C. The DNA precipitate was taken with a wide-bore pipette tip and washed with 500 µL of 70% ethanol, then centrifuged at low speed. After air-drying, DNA was stored at 4 °C in 67 µL ultrapure water. This DNA extract was used for the first sequencing run symbolized Run_1.

For the second sequencing run, symbolized Run_2, we collected 60 males from the same Horezu stock. DNA was extracted as described above.

A DNA concentration of 113.5 ng/µL was used in Run_1 and, respectively, a DNA concentration of 76 ng/µL in Run_2.

### 4.3. Nanopore Library Preparation, Sequencing, and Basecalling

The library preparation, sequencing and basecalling processes were performed according to the manufacturer’s protocol for the Rapid Sequencing kit (SQK-RAD004). In order to prepare the library, we mixed 7.5 μL genomic DNA with 2.5 μL FRA (Fragmentation Mix). After incubating the mixed DNA library at 30 °C for 1 min and then at 80 °C for 1 min, we added 1 μL of RAP (Rapid Adapter) in order to attach the sequencing adapters to the DNA fragments ends. The DNA/FRA/RAP mixture was incubated for 5 min at room temperature. Prior to loading the library, the flow cell was set up using SQB (Sequencing Buffer), FLT (Flush Tether), FB (Flush Buffer) and LB (Loading Beads) solutions. After removing the air bubbles inside the flow cell, we loaded 800 μL of the priming mix (30 μL FLT + an FB tube) into the priming port and let it stand for 5 min. In a separate tube, we mixed 34 μL of SQB , 25.5 μL of LB , 4.5 μL ultrapure water and the DNA library (11 μL). The resulting 75 μL mix was loaded into the sequencing port (SpotON) of the MinION.

We used two FLO-MIN106 type flow cells. Run_1 started with 909 available pores and ran for approximately 48 h. Run_2 started with 1400 available pores and ran for 72 h.

We used the MinKNOW tool version 3.6.5 for data acquisition and for converting the raw data files represented by electrical signals (FAST5) into FASTQ files (basecalling). 

Both collections of ONT reads have been uploaded to SRA/NCBI, under accession numbers SRA/NCBI: SRX8215201 and SRA/NCBI: SRX17355721, respectively.

### 4.4. Computational Environment

Oxford Nanopore MinION sequencing device was connected to a computer equipped with 32 Gb DDR4 RAM, an i7-6500U processor, 500 Gb SSD and Linux Mint 20 operating system. Basecalling and assembly steps were performed on the same device.

### 4.5. Data Processing and Quality Control

We used the EPI2ME platform (Oxford Nanopore, Oxford, UK) for the analysis of ONT data. EPI2ME (accessed on 22 December 2021) is able to provide quality control of the data and splits reads into “pass” and “fail”, based on high/low quality scores of the reads.

To eliminate adapters, we used Porechop version 0.2.4 (accessed on 30 March 2020) [10], which aligns reads subsets to the sequences of all adapters specific to ONT sequencing methodology and removes the adapter sequences from the end of the reads if they are detected. Then, we filtered the reads according to the quality score with NanoFilt tool (accessed on 21 April 2020) [9], designed for reads obtained by nanopore sequencing. The processed reads were quality assessed with NanoPlot [9], an application for visualizing and processing long reads (accessed on 30 March 2020).

### 4.6. De Novo Assembly

De novo assembly step was performed in a Linux environment using the following assemblers: i. Flye, version 2.8.3 (accessed on 5 July 2021)—an application for assembling sequences generated by ONT and Pacific Biosciences (PacBio), which can be used for both bacterial and eukaryotic genomes [4]; ii. Canu version 2.1.1 (accessed on 4 August 2021), specialized for assembly of high-noise long sequences [6].

The Flye—Data set II assembly was submitted to GenBank/NCBI, accession number JANZWZ000000000.1.

### 4.7. Assembly versus the Reference Genome of D. melanogaster

The guided assembly was performed using the *D. melanogaster* r6.39 reference genome from FlyBase [40]. Reference scaffolds that could not be associated with any *D. melanogaster* chromosomes (or mitochondrial DNA) were removed. The following programs were used to perform guided assembly:

1. Minimap2 version 2.20 (accessed on 20 June 2021)—a bioinformatics application designed to align long ONT and PacBio reads to a reference sequence. The program quickly aligns the nucleotide sequences with each other to identify overlapped regions and aligns the reads to the reference genome [7].
2. NGMLR version 0.2.8 (accessed on 21 June 2021)—a bioinformatics tool able to map ONT reads to a large reference genome. NGMLR application provides quick and accurate nucleotide sequences alignments, taking into account both possible sequencing errors and genomic variations [8].
3. SAMtools version 1.7 (accessed on 20 June 2021)—a suite of programs dedicated to process high-throughput sequencing data [41].

### 4.8. Assessing the Quality of Generated Assemblies

The following tools were used for the qualitative evaluation of the generated assemblies:

1. QUAST-LG (accessed on 4 August 2021) is one of the best-known tools for evaluating the quality of de novo genome assemblies. The application can also be used with a reference genome and supports multiple assemblies at the same time, which makes it suitable for comparative analyses [11];
2. BUSCO version 5.2.2 (accessed on 3 December 2021) searches in de novo assemblies for highly conserved USCOs. We used the metazoa_odb10 database, which contains 954 USCOs likely to be present in many metazoan genomes [12];
3. Qualimap version 2.2.1 (accessed on 19 July 2021) is a Java application that allows qualitative evaluation of the assemblies resulting following reads alignment to a reference genome. Guided assembly data (BAM files) are used to obtain a qualitative report that includes graphs and statistical parameters of the assembly [24];
4. BAMstats version 1.25 (accessed on 20 July 2021) is a graphical interface program used to calculate mapping statistics of reads from a BAM file. This application provides an overview of the query/reference genome alignment quality [25].

As an additional quality evaluation, we examined the Horezu genotype for genes sequences integrity by comparing it to the sequences of 53 control genes drawn from the *D. melanogaster* r6.39 reference genome. The control gene set consisted of *γCOP* gene and a particular selection of 52 genes involved in the Toll and Imd-Jnk immune pathways. Sequences of these genes were downloaded from FlyBase [40] and aligned against our de novo assemblies using blastn (accessed on 6 June 2021) [13] in the Linux terminal.

In addition, we also used RepeatMasker version 4.1.2 (accessed on 27 October 2022), a popular software developed to quantify the NTs content in re-sequenced genomes and currently being the gold standard for this type of analysis [19]. The program was run using the alignment application rmblastn version 2.10.0+ and NTs consensus database Dfam 3.3. To identify and analyze the insertions of mdg1 retroelement in the *D. melanogaster* Horezu genotype, we used Genome ARTIST (GA) v2 software (accessed on 01 November 2022) [20]. Genome sequencing, preprocessing and data analysis were performed in the *Drosophila* laboratory of the Department of Genetics, Faculty of Biology, University of Bucharest.

## Figures and Tables

**Table 1:** Statistics results compiled by NanoPlot for raw FASTQ files generated by the two nanopore sequencing runs.

Statistics	Run_1	Run_2
Total number of FAST5 files	173	158
Total read number	688,560	629,570
Size (Gb)	5	4.1
The longest read length (nt)	121,786	112,857
Mean read length	3548	3171
Mean read quality	10.8	8.1

**Table 2:** Statistical parameters of Data set I and II obtained with NanoPlot.

Statistics	Data Set I	Data Set II
Size (Gb)	8.9	4.1
Total number of reads	1,317,857	590,406
Mean q	9.3	11.8
Mean read length	3298	3356
Longest read	121,786	98,982
Total number of bases	4,346,556,125	1,981,948,635

**Table 3:** QUAST-LG statistics for the de novo assemblies obtained with Canu and Flye using Data set I and Data set II.

Assembly Statistics	Canu Data Set I	Canu Data Set II	Flye Data Set I	Flye Data set II
No. of contigs	3202	3586	1348	1531
Largest contig	4,036,320	1,027,435	10,359,939	3,223,716
N50	256,290	121,999	3,373,574	492,599
NG50	479,257	160,502	3,475,578	502,738
Total length	192,838,120	164,407,780	148,574,057	138,855,691
Reference length	137,567,484			
Genome fraction (%)	94.8	89.7	91.4	86.5
No. of misassembled contigs	1392	1408	268	382
No. of fully unaligned contigs	493	247	124	99
No. of possible NTs	434	298	132	90

**Table 4:** A general analysis of the repetitive sequences performed with RepeatMasker on Canu and Flye assemblies of Horezu strain versus minimap/miniasm—ISO1 (adapted from RepeatMasker outputs).

	Canu Data Set I		Canu Data Set II		Flye Data Set I		Flye Data Set II		Minimap/Miniasm ISO1	
Bases Masked	51769568 bp		41861639 bp		20608100 bp		18111261 bp		21716093 bp	
	(26.83%)		(25.45%)		(13.83%)		(12.99%)		(16.47%)	
	No. of Elements	Percentage of Seq (%)	No. of Elements	Percentage of Seq (%)	No. of Elements	Percentage of Seq (%)	No. of Elements	Percentage of Seq (%)	No. of Elements	Percentage of Seq (%)
**Retro elements**	22,385	18.93	19,128	18.46	11,300	8.65	10,272	8.17	9689	12.49
**LINEs:**	8295	6.39	7017	6.36	4001	2.95	3636	2.84	3579	4.24
L2/CR1/Rex	1144	0.61	1001	0.63	774	0.55	734	0.55	648	0.53
R1/LOA/Jockey	1727	1.86	1434	1.84	677	0.63	609	0.60	819	1.26
R2/R4/NeSL	69	0.08	57	0.07	17	0.01	16	0.01	18	0.03
**LTR elements**	14,090	12.54	12,111	12.10	7299	5.70	6636	5.33	6110	8.25
BEL/Pao	2882	2.26	2528	2.19	1836	0.96	1649	0.90	1398	1.92
Ty1/Copia	772	0.74	671	0.75	283	0.26	265	0.24	252	0.40
Gypsy	10,436	9.54	8912	9.17	5180	4.48	4722	4.19	4460	5.93
**DNA transposons**	5301	1.39	4429	1.39	3178	1.01	2943	1.01	2951	1.12
hobo-Activator	286	0.07	239	0.07	158	0.04	147	0.05	204	0.07
Tc1-IS630-Pogo	1408	0.38	1098	0.33	929	0.31	850	0.29	930	0.40
PiggyBac	31	0.01	22	0.01	21	0.01	24	0.01	12	0.01
Other (Mirage, P-element, Transib)	2825	0.70	2439	0.75	1559	0.48	1433	0.47	1402	0.49
Rolling-circles	5689	0.63	5071	0.65	4456	0.64	4385	0.67	3213	0.53
**Unclassified**	473	0.04	374	0.03	372	0.04	301	0.04	320	0.04
**Small RNA**	1061	0.41	761	0.36	289	0.06	169	0.04		
**Total interspersed repeats**		20.35		19.89		9.70		9.22		13.65
**Satellites**	1602	1.40	1280	0.95	735	0.52	598	0.38	739	0.34
**Simple repeats**	85,262	3.79	76,658	3.34	81,534	2.60	75,898	2.37	50,764	1.64
**Low complexity**	9737	0.25	8827	0.25	9777	0.31	9155	0.31	6613	0.25

**Table 5:** Insertions of mdg1 in Canu—Data set I, Canu—Data set II, Flye—Data set I, Flye—Data Set II and minimap/miniasm—ISO1 genome assemblies reported by GA_v2. Insertions found in both a specific assembly and the *D. melanogaster* reference genome (r6.48) are conserved, while those found only in Horezu strain are specific. An insertion that was mapped at chromosome level with an acceptable margin of error is considered ambiguous. Unresolved insertions could not be mapped because of the repetitive nature of flanking sequences. Only resolved insertions were considered when counting the total number of mapped insertions.

Type of mdg1 Insertion	Canu Data Set I	Canu Data Set II	Flye Data set I	Flye Data Set II	Minimap/Miniasm ISO1
**Conserved**	10	10	7	6	17
**Horezu specific**	29	28	3	4	-
**Ambiguous**	5	-	1	1	-
**Unresolved**	7	6	3	1	1
**Total mapped insertions**	**44**	**38**	**11**	**11**	**17**

**Table 6:** Statistical parameters obtained with Qualimap for the quality of the guided assemblies performed with the minimap2 and NGMLR applications using the two datasets.

Statistics	Minimap2 Data Set I	Minimap2 Data Set II	NGMLR Data set I	NGMLR Data Set II
Mapped reads (%)	90.27	96.45	73.93	84.56
Mean Coverage	26.52	13.51	21.45	11.15
Mean Mapping Quality	50.4	51.08	47.1	48.82
General error rate (%)	15.82	11.76	12.85	8.69

## Data Availability

Horezu strain sequencing project—SRA/NCBI: SRX8215201, Horezu sequencing—Run: SRR11654246, Horezu re-sequencing—SRA/NCBI: SRX17355721, Run: SRR21349872. Draft Horezu Genome Assembly (Flye—Data set II) was uploaded under GenBank/NCBI accession JANZWZ000000000.1.

## Supplementary Materials

The following supporting information can be downloaded at: https://www.mdpi.com/article/10.3390/ijms232314892/s1, Figure S1: Immune-system related genes identified with BLAST in Canu—Data set I assembly, Table S1: Genes associated with Toll, Imd and Imd-JNK pathways, used for BLAST screening against Canu and Flye contigs, Table S2: BLAST results for immune related genes identified in Horezu genotype, Tables S3–S6: Mapping of mdg1 NT in Horezu strain of *D. melanogaster* relative to the reference genome (r6.48), Table S7: Mapping of mdg1 NT in ISO1 strain of *D. melanogaster*, Table S8: Coverage values and number of reads covering each chromosome inferred for the guided assemblies using minimap2 and NGMLR applications.
