# Supplementary material for: ONT-Based Alternative Assemblies Impact on the Annotations of Unique versus Repetitive Features in the Genome of a Romanian Strain of Drosophila melanogaster
Source: Int J Mol Sci. 2022 Nov 28;23(23):14892. doi: 10.3390/ijms232314892 (PMC9741293; doi:10.3390/ijms232314892)
Supplement: Supplementary file 1 [file ijms-23-14892-s001.zip › ijms-1964632_Suppl_Figure_S1.pdf]

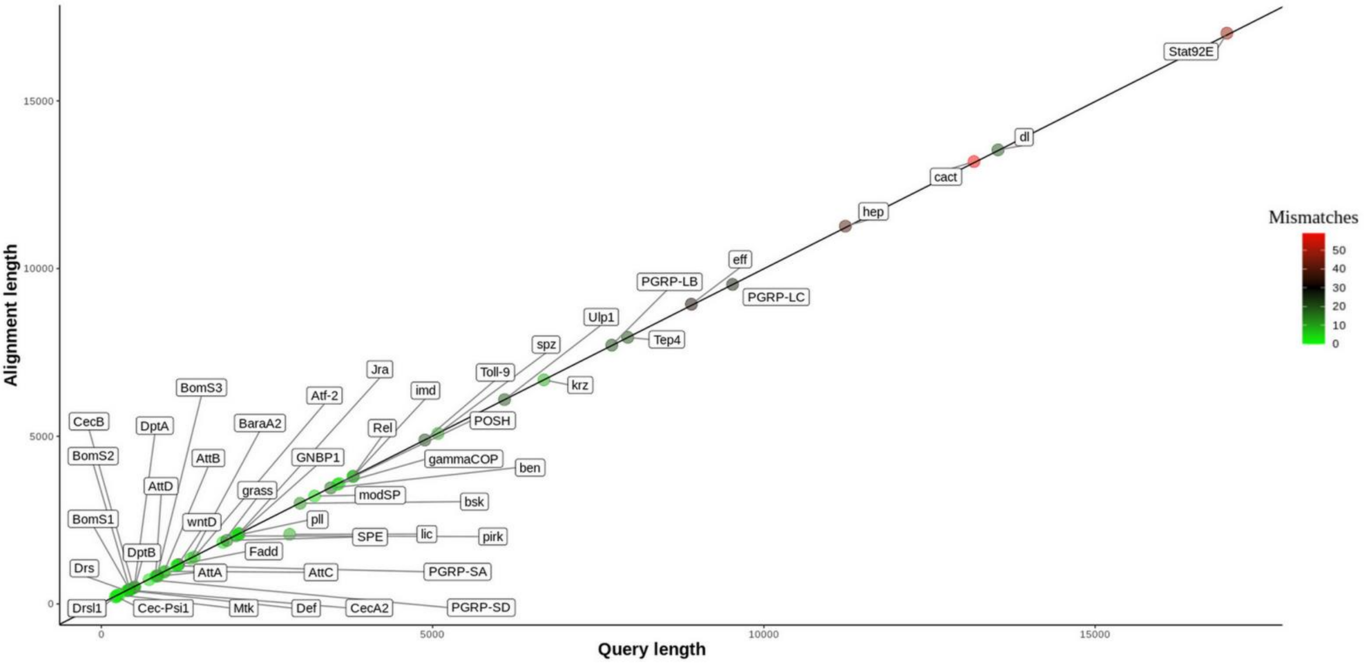

Figure S1. Immune-system related genes identified with BLAST in *Horezu_LaPeri* genome assembly obtained with the Canu and Data set I. The graph is showing a near perfect positive correlation (r = 0.999) between the query sequence length (*x* axis) of each gene and the alignment length (*y* axis). The numbers next to the color legend represent the identified mismatches when aligning the reference sequence of the genes with the Canu - Data Set I assembly. Summarizing, we identified a number of 6 genes with zero mismatches and 99% identity score (*AttD*, *DptA*, *Drsl1*, *Mtk*, *pll*, and *wntD*) and 24 genes with 1-9 mismatches and an identity score between 93-99% (*PGRP-SA*, *Drs*, *grass*, *Rel*, *AttB*, *γCOP*, *modSP*, *PGRP-SD*, *POSH*, *BomS1*, *Def*, *Atf-2*, *AttA*, *BaraA2*, *GNBP1*, *AttC*, *Cec-Psi1*, *Jra*, *BomS2*, *CecB*, *krz*, *lic*, *pirk*, *rel* and *spz*). The rest of the genes (*BomS3*, *bsk*, *DptB*, *SPE*, *CecA2*, *ben*, *dl*, *Toll-9*, *Ulp1*, *imd*, *PGRP-LB*, *Tep4*, *PGRP-LC*, *eff*, *Fadd*, *hep*, *Stat92E*, and *cact*) had a number of mismatches between 10 and 59 with an identity score of 94-99%. Apart from these, *Cec2* exhibited only 87% percentage identity score and was fully aligned but with 10 mismatches. This lowest alignment score is caused by the small size of the gene (240 bp) and the 10 mismatches detected following BLAST alignment. Also, the *Oamb*, *Plc21C* and *Gprk2* genes have been identified with a significant alignment score but due to their large size (> 25,560 bp) the BLAST results are fragmented. *Oamb*, *Plc21C* and *Gprk2* genes have not been included in the plot or data table.
