# Supplementary material for: ONT-Based Alternative Assemblies Impact on the Annotations of Unique versus Repetitive Features in the Genome of a Romanian Strain of Drosophila melanogaster
Source: Int J Mol Sci. 2022 Nov 28;23(23):14892. doi: 10.3390/ijms232314892 (PMC9741293; doi:10.3390/ijms232314892)
Supplement: Supplementary file 1 [file ijms-23-14892-s001.zip › ijms-1964632_Suppl_Table_S1.pdf]

Table S1. Genes associated with Toll, Imd and Imd-JNK pathways, used for BLAST screening against Canu and Flye contigs.

Gene name	Gene symbol
Activating transcription factor-2	*Atf-2*
Attacin-A	*AttA*
Attacin-B	*AttB*
Attacin-C	*AttC*
Attacin-D	*AttD*
Baramicin A2	*BaraA2*
basket	*bsk*
bendless	*ben*
Bomanin Short 1	*BomS1*
Bomanin Short 2	*BomS2*
Bomanin Short 3	*BomS3*
cactus	*cact*
Cecropin 2	*Cec2*
Cecropin A2	*CecA2*
Cecropin B	*CecB*
Cecropin pseudogene 1	*Cec-Ψ1*
Defensin	*Def*
Diptericin A	*DptA*
Diptericin B	*DptB*
dorsal	*dl*
Drosomycin	*Drs*
Drosomycin-like 1	*Drsl1*
effete	*eff*
Fas-associated death domain	*Fadd*
G protein-coupled receptor kinase 2	*Gprk2*
γCOP	*γCOP*
Gram-negative bacteria binding protein 1	*GNBP1*
Gram-positive Specific Serine protease	*grass*
hemipterous	*hep*
Immune deficiency	*imd*
Jun-related antigen	*Jra*
kurtz	*krz*
licorne	*lic*
Metchnikowin	*Mtk*
modular serine protease	*modSP*
Octopamine receptor in mushroom bodies	*Oamb*
pelle	*pll*
Peptidoglycan recognition protein LB	*PGRP-LB*
Peptidoglycan recognition protein LC	*PGRP-LC*
Peptidoglycan recognition protein SA	*PGRP-SA*
Peptidoglycan recognition protein SD	*PGRP-SD*
Phospholipase C at 21C	*Plc21C*
Plenty of SH3s	*POSH*
poor Imd response upon knock-in	*pirk*
Relish	*Rel*
Signal-transducer and activator of transcription protein at 92E	*Stat92E*
spatzle	*spz*
Spatzle-Processing Enzyme	*SPE*
spheroide	*sphe*

Thioester-containing protein 4	*Tep4*
Toll-9	*Toll-9*
Ulp1	*Ulp1*
wnt inhibitor of Dorsal	*wntD*
