# Supplementary material for: ONT-Based Alternative Assemblies Impact on the Annotations of Unique versus Repetitive Features in the Genome of a Romanian Strain of Drosophila melanogaster
Source: Int J Mol Sci. 2022 Nov 28;23(23):14892. doi: 10.3390/ijms232314892 (PMC9741293; doi:10.3390/ijms232314892)
Supplement: Supplementary file 1 [file ijms-23-14892-s001.zip › ijms-1964632_Suppl_Table_S3.pdf]

Table S3. Mapping of mdg1 NT in Horezu strain of *D. melanogaster* (Canu – Data set I) relative to the reference genome (r6.48).

Contig	Insertions Present in *D. melanogaster* r6.48	Insertions Specific for Horezu strain	Hit Genes
118	-	22053669, 2R	-
219	unannotated mdg1, 3709286, 3R	-	-
426	-	22341885, 3L	*Ten-m*
441	-	6140484, 3R	-
465	-	14237075, 3R	idefix{}1360<br>*CG43291*
537	-	969191, 2R	-
578	unannotated mdg1, 3061495,3R	-	*Pzl*
597	mdg1{}6444, Y	-	-
613	-	12110722, 2L	-
670, 2284	-	10679091, 2R	*stan*
712, 2325, 2326, 3480	unannotated mdg1, 3732178, 2R	-	-
789	-	13108934, 2R	-
834	-	1158046, X	-
853, 2571, 2692, 3396	unannotated mdg1, 23349629, X	-	-
863	-	2759484, Y	-
917	-	23901390, 3R	*sba*
1655	-	14364087, 3L	*fz*
2020	-	27095755, 3R	*CG14259*
2024	-	23213223, 2R	-
2063	-	14256790, 3L	-
2218	-	9082253, 3R	*pum*
2245	-	5165935, 3R	accord2{}625
2253	-	11717484, 3R	-
2285	-	21670273, X	gypsy{}2118
2466	-	11697217, 3R	*CG14717*
2467	-	230059, 2R	-
2665	-	10904411, 3R	-
2666	-	1158046, X	-
2674	-	5578671, 2L	-
2689	unannotated mdg1, 2775804, Y	-	-
2724	unannotated mdg1, 3746519, 2R	-	-
2817, 2818	most probably in Unmapped Scaffold 8		
2853	-	10786235, 3R	*Rbp1*
2854		most probably in X	
3030, 3662	unannotated mdg1, 2785427, Y	-	-
3230	-	891223, Y	-
3264	-	2517542, Y	-
3273, 3274, 3275		most probably in Y	
3348, 3349	unannotated mdg1, 2394302, Y	-	*WDY*
3386		most probably in 2R	
3395		most probably in X	
3397	-	11835, 2R	-
3535	-	23376272, X	-
3724	unannotated mdg1, 2384651, Y	-	*WDY*

For Canu – Data set I assembly we successfully mapped 44 mdg1 insertions. Ten mdg1 copies are preserved between the Horezu genotype and *D. melanogaster* r6.48; two of them are located in *WDY* gene and another copy is located in *Pzl* gene. Of these ten insertions, only one is currently annotated in the reference genome. Twenty-nine mdg1 copies are present only in Horezu genotype and inserted in *Ten-m*, *CG43291*,

stan, *sba*, *fz*, *CG14259*, *pum*, *CG14717* and *Rbp1* genes. The remaining five mdg1 insertions are considered ambiguous and they were mapped at the chromosome level.
