# Supplementary material for: ONT-Based Alternative Assemblies Impact on the Annotations of Unique versus Repetitive Features in the Genome of a Romanian Strain of Drosophila melanogaster
Source: Int J Mol Sci. 2022 Nov 28;23(23):14892. doi: 10.3390/ijms232314892 (PMC9741293; doi:10.3390/ijms232314892)
Supplement: Supplementary file 1 [file ijms-23-14892-s001.zip › ijms-1964632_Suppl_Table_S4.pdf]

Table S4. Mapping of mdg1 NT in Horezu strain of *D. melanogaster* (Canu – Data set II) relative to the reference genome (r6.48).

Contig	Insertions Present in *D. melanogaster* r6.48	Insertions Specific for Horezu strain	Hit Genes
35	unannotated mdg1, 2394302, Y	-	*WDY*
51	-	27095755, 3R	*CG14259*
94, 2864	unannotated mdg1, 2068089, 3R	-	*Myo81F*
115	-	10904410, 3R	-
143	-	969191, 2R	-
253	-	12110726, 2L	-
276	-	12307371, 3R	-
351	unannotated mdg1, 717426, 2R	-	-
355	-	14576524, 2L	*lncRNA:CR44856*
454	-	10679036, 2R	*stan*
522, 2618, 3262	unannotated mdg1, 23349609, X	-	-
592, 2719	unannotated mdg1, 3732166, 2R	-	-
930	-	22969915, 3L	-
968	-	20897650, 3L	*CG43931*
988	unannotated mdg1, 2775806, Y	-	-
1164	-	1158046, X	-
1226	-	9082267, 3R	*pum*
1234	-	2759484, Y	-
1365	-	10652704, X	*X11LBeta*
2231	unannotated mdg1, 3061493, 3R	-	*Pzl*
2253	-	14364087, 3L	*fz*
2298	-	5165951, 2R	accord2{}625
2865	unannotated mdg1, 2036796, 3R	-	*Myo81F*
2976	unannotated mdg1, 3709286, 3R	-	-
3146, 3314, 4095	-	23901390, 3R	*sba*
3147	-	22053669, 2R	-
3289	-	267968, 2R	-
3290	-	14256789, 3L	-
3684	unannotated mdg1, 3470437, Y	-	-
3687	-	32009946, 3R	*heph*
3744	-	891223, Y	-
3744	-	2653795, Y	-
3762	-	1415939, Y	-
3820	-	23213233, 2R	-
4086	-	20498, 2R	-
4122	-	24285757, 3L	*Snap25*
4179	-	20332573, 3R	-
4180	-	680342, X	-

For Canu – Data set II assembly we mapped 38 mdg1 insertions. Twenty-eight mdg1 copies are present only in Horezu genotype and inserted in 10 genes, namely *CG14259*, *lncRNA:CR44856*, *stan*, *CG43931*, *pum*, *X11LBeta*, *fz*, *sba*, *heph* and *Snap25*. The remaining 10 mdg1 copies were found in both *D. melanogaster* r6.48 and Horezu; two of them are located in *Myo81E* gene, and another two are located in *WDY*, respectively *Pzl* genes.
