# Supplementary material for: ONT-Based Alternative Assemblies Impact on the Annotations of Unique versus Repetitive Features in the Genome of a Romanian Strain of Drosophila melanogaster
Source: Int J Mol Sci. 2022 Nov 28;23(23):14892. doi: 10.3390/ijms232314892 (PMC9741293; doi:10.3390/ijms232314892)
Supplement: Supplementary file 1 [file ijms-23-14892-s001.zip › ijms-1964632_Suppl_Table_S5.pdf]

Table S5. Mapping of mdg1 NT in Horezu strain of *D. melanogaster* (Flye – Data set I) relative to the reference genome (r6.48).

Contig	Insertions Present in *D. melanogaster* r6.48	Insertions Specific for Horezu strain	Hit Genes
22	-	23901390, 3R	*sba*
23	unannotated mdg1, 2775818, Y	-	-
601	unannotated mdg1, 3061511, 3R	-	*Pzl*
656	unannotated mdg1, 2012000, 3R	-	*Myo81F*
656	unannotated mdg1, 2073227, 3R	-	*Myo81F*
800		Most probably 2R	
1486	mdg1{}799, 2R	-	-
1529	unannotated mdg1, 951754, 2R	-	-
1610	unannotated mdg1, 3709264, 3R	-	-
1707	-	21670273, X	-
1727	-	1158046, X	-

For the Flye – Data set I assembly we mapped 11 mdg1 insertions. Seven copies are present in the Horezu genotype and *D. melanogaster* r6.48; a single copy is annotated in the reference genome and two unannotated copies are located in *Pzl* and *Myo81E* genes. Three identified insertions are specific to the Horezu genotype, and of these, only one is inserted in *sba* gene. We've identified a single ambiguous insertion in chromosome 2R.
