# Supplementary material for: ONT-Based Alternative Assemblies Impact on the Annotations of Unique versus Repetitive Features in the Genome of a Romanian Strain of Drosophila melanogaster
Source: Int J Mol Sci. 2022 Nov 28;23(23):14892. doi: 10.3390/ijms232314892 (PMC9741293; doi:10.3390/ijms232314892)
Supplement: Supplementary file 1 [file ijms-23-14892-s001.zip › ijms-1964632_Suppl_Table_S6.pdf]

Table S6. Mapping of mdg1 NT in Horezu strain of *D. melanogaster* (Flye – Data set II) relative to the reference genome (r6.48).

Contig	Insertions Present in *D. melanogaster* r6.48	Insertions Specific for Horezu strain	Hit Genes
98	-	10652704, X	*X11LBeta*
533	unannotated mdg1, 3061509, 3R	-	*Pzl*
555		Most probably 2R	
647	mdg1{}6544, Y		-
678	-	14256789, 3L	-
688	unnanotated mdg1, 3709260, 3R	-	-
800	-	32009946, 3R	*heph*
1143	unannotated mdg1, 951758, 2R	-	-
1399	-	21670273, X	gypsy{}2118
1406	unannotated mdg1, 2387578, Y	-	*WDY*
2464	unannotated mdg1, 3745902, 2R	-	-

For the Flye – Data set II assembly we mapped 11 mdg1 copies. Six of these are preserved between the Horezu genotype and *D. melanogaster* r6.48, but only one is annotated. Two of the six conserved copies are located in *Pzl*, respectively *WDY* genes. We've identified four Horezu specific insertions and two of them are inserted in *X11LBeta*, respectively *heph* genes. We found a single ambiguous insertion in chromosome 2R.
