# Supplementary material for: ONT-Based Alternative Assemblies Impact on the Annotations of Unique versus Repetitive Features in the Genome of a Romanian Strain of Drosophila melanogaster
Source: Int J Mol Sci. 2022 Nov 28;23(23):14892. doi: 10.3390/ijms232314892 (PMC9741293; doi:10.3390/ijms232314892)
Supplement: Supplementary file 1 [file ijms-23-14892-s001.zip › ijms-1964632_Suppl_Table_S7.pdf]

**Table S7.** Mapping of mdg1 NT in ISO1 strain of *D. melanogaster* relative to the reference genome (r6.48).

| Contig | Insertions Present in *D. melanogaster* r6.48 | Insertions Specific for Minimap ISO1 strain | Hit Genes |
|---|---|---|---|
| utg000001l | mdg1{}299, 2L | - | - |
| utg000001l | mdg1{}305, 2L | - | *dpy* |
| utg000002l | mdg1{}CG42335[iso-1], 3R | - | *CG42335* |
| utg000002l | mdg1{}1280, 3R | - | *Teh1* |
| utg000004l | mdg1{}1047, 3L | - | *CG14082* |
| utg000011l | mdg1{}914, 3L | - | - |
| utg000028l | mdg1{}831,2R | - | *hbs* |
| utg000033l | mdg1{}2138, X | - | *lncRNA:flam* |
| utg000034l | mdg1{}885, 2R | - | *Mid1* |
| utg000035l | mdg1{}29, X | - | *Kirre* |
| utg000041l | unannotated mdg1, 4300896, 2R | - | - |
| utg000041l | mdg1{}6612, 2R | - | *Gprk1* |
| utg000047l | mdg1{}1442, 3R | - | *CG34354* |
| utg000055l | mdg1{}1610, mdg1{}1023, 3L | - | *Ccn* |
| utg000064l | mdg1{}6543, 3R | - | *Parp* |
| utg000074l | mdg1{}1678, 2R | - | - |
| utg000074l | mdg1{}1720, 2R | - | *CG11883* |

For the Minimap ISO1 assembly we mapped 17 mdg1 copies. Sixteen copies are annotated and located in 13 genes, *dpy*, *CG42335*, *Teh1*, *CG14082*, *hbs*, *lncRNA:fam*, *Mid1*, *Kirre*, *Gprk1*, *CG34354*, *Ccn*, *Parp* and *CG11883*. We identified a single case of an unannotated mdg1 copy in the reference genome.
