# Supplementary material for: ONT-Based Alternative Assemblies Impact on the Annotations of Unique versus Repetitive Features in the Genome of a Romanian Strain of Drosophila melanogaster
Source: Int J Mol Sci. 2022 Nov 28;23(23):14892. doi: 10.3390/ijms232314892 (PMC9741293; doi:10.3390/ijms232314892)
Supplement: Supplementary file 1 [file ijms-23-14892-s001.zip › ijms-1964632_Suppl_Table_S8.pdf]

Table S8. Coverage values and number of reads covering each chromosome inferred for the guided assemblies resulted by using Data set I and Data set II with both minimap2 and NGMLR applications (source: BAMstats).

Reference sequence	No. aligned reads	Mean coverage	Input Data
2L	286,714	36.61	Minimap2 Data set I
2R	383,793	41.55	
3L	415,962	40.23	
3R	432,369	36.33	
4	26,035	44.58	
X	440,673	33.21	
Y	264,826	119.63	
mitochondrion	1,344	102.75	
2L	150,217	19.10	Minimap2 Data set II
2R	201,513	21.74	
3L	214,554	21	
3R	218,340	18.25	
4	14,392	24.86	
X	202,525	17.06	
Y	135,499	67.48	
mitochondrion	806	55.29	
2L	188,301	23.40	NGMLR Data set I
2R	210,587	23.20	
3L	231,301	23.17	
3R	303,209	23.80	
4	17,048	23.46	
X	136,236	13.23	
Y	80,271	15	
mitochondrion	975	78.65	
2L	99,646	12.19	NGMLR Data set II
2R	109,218	11.90	
3L	122,463	12.23	
3R	166,959	12.24	
4	11,012	13.17	
X	69,730	6.72	
Y	54,855	9.14	
mitochondrion	587	44.28	
